# Purchasing equipment for an eye unit

**Published:** 2010-09

**Authors:** Catherine Cross, Philip Hoare

**Affiliations:** Formerly Manager, International Programmes, Sightsavers, Grosvenor Hall, Bolnore Road, Haywards Heath, West Sussex, RH16 4BX, United Kingdom.; Programme Procurement Manager, Sightsavers, Grosvenor Hall, Bolnore Road, Haywards Heath, West Sussex, RH16 4BX, United Kingdom.

**Figure:**
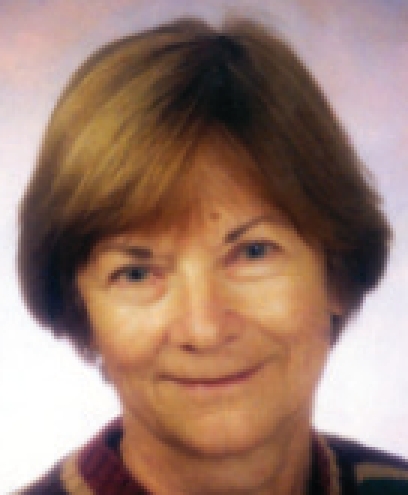


**Figure:**
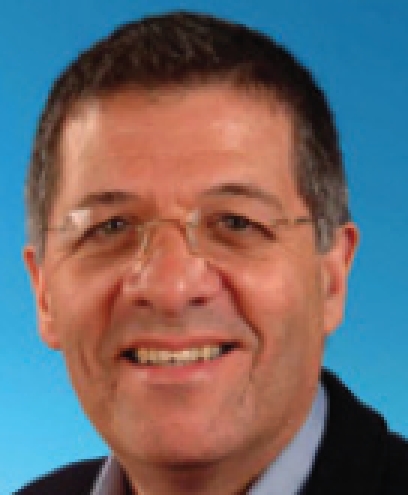


If VISION 2020 is to meet its objective of eliminating avoidable blindness in ten years' time, eye units have to ensure that they have the equipment they need so they can work quickly and effectively. In this article, we aim to show you how to get the best results when purchasing (procuring) equipment.

## Who is responsible?

The manager of the eye care unit is ultimately responsible for ensuring that equipment and supplies are adequate.

However, the manager may delegate a member of the team to act as the ‘equipment person’. This person will have some responsibilities for ordering and will also be responsible for keeping the **equipment inventory** up to date. The inventory should contain information about all items of equipment in the eye unit: when these were bought, from whom, at what cost, and where the items are now situated in the hospital or clinic. When new equipment arrives, this should also be recorded in the inventory. The equipment inventory can be stored on computer or in a paper filing system.

The equipment person will also be responsible for ensuring that all equipment has sufficient consumables (such as reagents, filters, recording paper), spare parts (including light bulbs) and accessories (cables, electrodes, and so on) and will order replacements when stocks run low.

The equipment inventory is a good place to note how satisfied the eye unit is with the services offered by different suppliers. When it is time to order again, this information will help the equipment person to determine whether to continue with the same supplier or consider other suppliers.

## Finding out what you need

The *IAPB Standard List fora VISION 2020 Eye Care Service Unit* (Standard List) is a useful guide to the equipment you are likely to need in your eye unit. The Standard List is updated every two years by the IAPB Technology Programme Committee and also contains the names and contact details of suppliers (see page 36 for details on how to get your own copy).

With equipment, you have to be sure that what you buy will last and will suit your needs and circumstances. In addition to consulting the Standard List, we would recommend that you also obtain catalogues or visit suppliers' websites to see what is available and whether it meets your needs.

It is helpful to ask yourself the following questions about the equipment you want to buy:

- Is the item durable and can it handle heavy use?
- Is the model already in use locally and is there local expertise to repair and maintain it?
- If other local eye units are using the same or similar equipment, have they had good results, or encountered problems with the functioning or maintenance of the equipment?
- What is the cost of the item?
- What is the cost of accessories, consumables, and spare parts, and are they available in our country?
- What are the associated requirements (electrical consumption, wiring, water supply, environment, room temperature) and can we provide these? What electric voltage does the item require and is it compatible with what we have?
- If the local electricity supply is erratic, will back-up items such as electrical stabilisers and voltage regulators also be necessary? What are the costs?
- Is training needed in the use and care of the equipment? Who can reliably conduct the training? What is the likely cost?
- Will it be necessary for the supplier to come and install the equipment and/or maintain it? Will special training in use and maintenance be needed?

**NOTE:** The article about donated equipment on page 32 contains some additional questions you can discuss with the supplier before acquiring new equipment. Most of these are also valid for purchased equipment.

## Budgeting for an equipment purchase

Prices quoted in a catalogue or on the internet may not include value added (or sales) tax. As this varies from country to country, you may have to ask the supplier what the full cost is.

If the equipment is to be imported, the cost of packing, insurance, and clearing must be included in your budget.

As mentioned above, you also need to budget for accessories, spare parts, consumables, maintenance, user training, and electrical stabilisers, if needed. As a guide, budget 3-6% of the purchase price for every year of use. It is recommended that you order enough consumables and spare parts for at least one year when purchasing the equipment.

**Figure:**
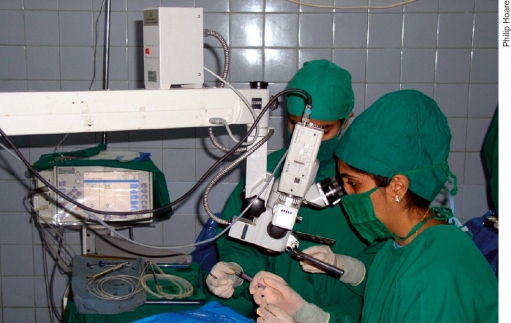
When buying expensive equipment such as an operating microscope, ensure that you have also budgeted for accessories, spare parts, consumables, user training, and electrical stabilisers such as voltage regulators (if needed). BANGLADESH

Philip Hoare

**Figure:**
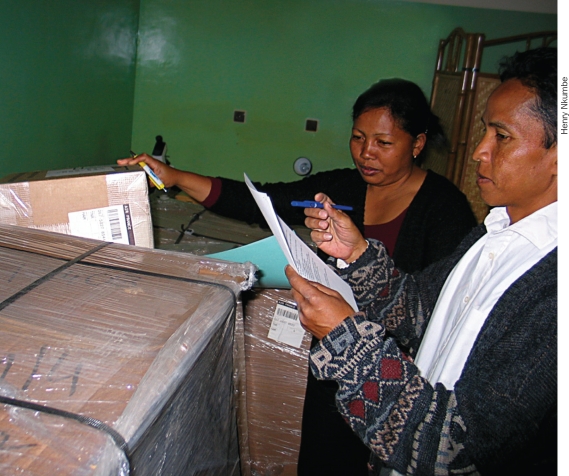
Members of the eye care team check the paperwork of newly arrived eye care equipment and consumables. They will also check the integrity of the boxes to ensure they haven't been tampered with en route. MADAGASCAR

Henry Nkumbe

## Finding a supplier

Once you know what you need, look for the best supplier (if you are satisfied with your existing suppliers, you can skip this step). The Standard List is a good place to start - it contains the names and contact details of suppliers for all of the equipment and consumables it lists.

Once you have identified suppliers who can provide what you need, request up-to-date catalogues or price lists as well as more information about their products. You can ask for quotations, or invite the supplier to tender or submit a proposal.

When planning to buy expensive items, in particular, you may want to evaluate the supplier before making a decision. Ask the supplier whether they can put you in touch with some of their other customers. Suppliers who are confident in their products and services will normally be happy to do so.

You may ask to examine samples of the product being considered, or ask for a trial period with an item of equipment. At least request to see a photograph of the equipment - this will help ensure that there hasn't been a misunderstanding about what exactly you want to buy!

Depending on the product, ask whether installation, warranty, and maintenance (or a short maintenance workshop for local staff) are included in the price.

This is also a good time to negotiate access to the **service manual** (see tip on page 31), particularly if your eye unit is in a remote location and specialised maintenance and repair personnel are not available.

Before making a final decision, ask the supplier the following:

- Do they supply the consumables and spare parts as well, or will you need to go elsewhere?
- Will they help to set up the equipment once it arrives?
- What services do they offer if the equipment breaks down, and will they send a replacement while yours is being repaired?
- Will they train local staff in maintenance and replacement of spare parts?

## Preparing an order

The more details that are included on the order, the greater the likelihood of a speedy response from the supplier and of receiving exactly what was requested. Include:

- Catalogue number
- Make and model
- Detailed description of item
- Quantity
- Unit cost
- Total cost

The catalogue or supplier's website should be used to ensure that the description and specifications are correct.

**NOTE:** Remember to order sufficient spare parts for the first year, such as spare microscope or operating light bulbs

**‘When planning to buy expensive items, you may want to evaluate the supplier before making a decision’**

The ‘Operating Room’ section of the Standard List helps by placing these, with references, under the main item. Ask the supplier what spare parts maybe needed in the first year, such as light bulbs, fuses, spare paper for an A-scan, and fungicidal (anti-mould) tablets; these should be ordered at the same time.

## Placing the order and paying for it

Most overseas suppliers will require payment in advance or with order. The majority of the suppliers in the Standard List are tried and tested, but be cautious when dealing with unknown suppliers. You can use an inspection company such as SGS^1^ or Cotecna^2^ to verify such transactions and ensure your equipment is shipped as contracted.

It is vital to give the correct shipping details (in particular, your correct address) to the suppliers. This information will be used to ship your equipment and will be applied to all shipping documentation. If the details are incorrect it could delay the shipment and/or clearance through customs, which may result in an expensive demurrage charge (a charge applied when shipped goods are not collected on time).

The local authorities may allow you to import duty free. In such cases, a gift certificate or certificate of donation might have to accompany the shipment. At this stage, it is worth checking with the local authorities whether any other documentation is required to import your equipment, such as an inspection certificate or certificate of origin.

## Receiving the order

Prompt clearance at the port or airport is important. This will reduce extra charges (demurrage) and minimise the chances of loss or damage. The member of your team responsible for clearance will need to be familiar with the clearance procedures, including any customs and duty waiver. When placing the order with an overseas supplier, insist as part of the contract that they give you the full shipping details before shipping the equipment. This will allow you to make the necessary plans to clear the goods well in advance of the date of shipment. The equipment person should check the goods received against the order immediately on arrival. Are the correct items received, in the right quantity? Is the quality good and is the equipment functioning as required? Delays in checking incoming goods may result in the guarantee running out before faults are discovered.

The equipment person should enter items received into the equipment inventory with details of date of purchase, model, serial number, and cost. All associated documents (including manuals, service contracts, and warranties) should be filed in a safe place.

## Setting up and testing equipment

Ideally, this should be done by the supplier's agent or a medical equipment engineer, but members of the eye care team should participate in order to learn how the equipment works and how to care for it. Where there is no medical equipment engineer or supplier's agent available, it is important that the item is carefully unpacked and the manual thoroughly studied before assembly.

**Figure:**
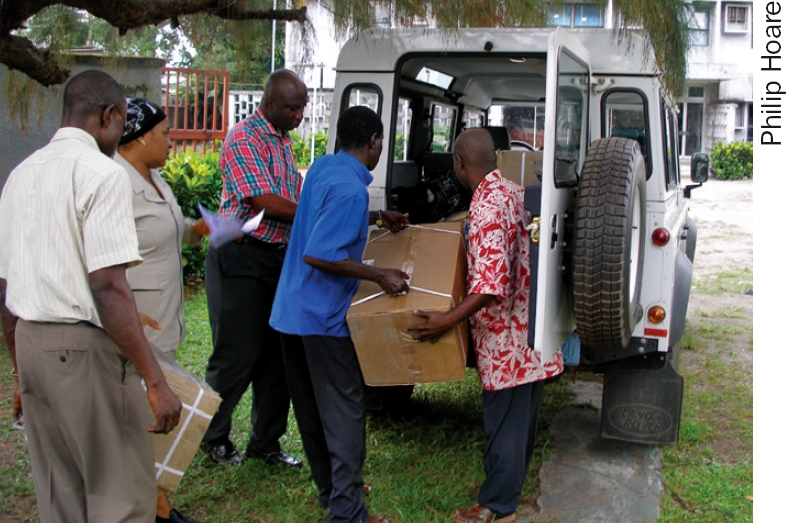
Take care when transporting new equipment. NIGERIA

Philip Hoare

Testing the equipment needs to be done thoroughly, particularly in the case of electrical items. The connections and voltage must be checked and voltage stabilisers and regulators installed in areas where electrical supply is unreliable.

In conclusion, if procurement is approached in a planned, systematic way, as described in this article, you will have the best possible chance to acquire good quality equipment that will meet your needs well into the future.
